# Iridescence impairs object recognition in bumblebees

**DOI:** 10.1038/s41598-018-26571-6

**Published:** 2018-05-25

**Authors:** Karin Kjernsmo, Joanna R. Hall, Cara Doyle, Nadia Khuzayim, Innes C. Cuthill, Nicholas E. Scott-Samuel, Heather M. Whitney

**Affiliations:** 1 School of Biological Sciences, University of Bristol, Bristol, BS8 1TQ UK; 2 School of Experimental Psychology, University of Bristol, Bristol, BS8 1TU UK

## Abstract

Iridescence is a taxonomically widespread and striking form of animal coloration, yet despite advances in understanding its mechanism, its function and adaptive value are poorly understood. We test a counterintuitive hypothesis about the function of iridescence: that it can act as camouflage through interference with object recognition. Using an established insect visual model (*Bombus terrestris*), we demonstrate that both diffraction grating and multilayer iridescence impair shape recognition (although not the more subtle form of diffraction grating seen in some flowers), supporting the idea that both strategies can be effective means of camouflage. We conclude that iridescence produces visual signals that can confuse potential predators, and this might explain the high frequency of iridescence in many animal taxa.

## Introduction

Iridescence is a form of structural coloration caused by interference of light reflected from nanostructures within a surface^1^. Iridescence can be generated though a wide range of mechanisms, including thin-film interference, diffraction gratings, multilayers and photonic crystals^1–6^. However, the two most common mechanisms underlying iridescence in both animals and plants are multilayers and diffraction gratings^7^ and in all cases, the result of these optical phenomena is that the hue and intensity of the reflected light will vary depending on the angle of view or angle of illumination, as illustrated by the shimmering fruits of *Pollia condensata*, vivid green and orange breast feathers of the male Lawes’s parotia (*Parotia lawesii*), a bird of paradise, the green and purple stripes of the Japanese jewel beetle (*Chrysochroa fulgidissima*), and the brilliant wings of the blue morpho butterflies (*Morpho* sp.)^6–9^.

Due to its unique optical properties and striking appearance, iridescence in animals is, understandably, usually assumed to be a signal^2^. In species with strong sexual dimorphism, such as the Indian peafowl (*Pavo cristatus*), iridescence may serve an important function in mate choice^2,4,10–12^. However, it is also common in monomorphic animals, such as beetles, and in non-reproductive life stages such as butterfly chrysalises^2,3^. Here, the evolution of iridescence by sexual selection is a priori unlikely. Instead, the American artist and father of modern camouflage theory, Abbot Thayer, proposed that iridescence was an anti-predator adaptation. Thayer described his observations of iridescent insects: “*Brilliantly changeable or metallic colors are among the strongest factors in animals’ concealment*”^13^ (p.87). He continued: “*Even without motion, the animal’s surface, which would show all in its true place and plane if it were plainly colored, is by its iridescence made to appear ‘dissolved’ into many depths and distances*”.

Thayer’s statement is counter-intuitive: how can something that is both brilliant and changeable contribute to concealment? Camouflage generally works through matching the background, mimicking inedible objects (masquerade), or by utilizing colour patterns that break up the otherwise recognisable shape of a prey (disruptive coloration)^14,15^. The latter may impede identification by predators searching for a typical shape: even if individual colour patches are detected, they are not recognised as belonging to a single object and so the prey remains unrecognised^13,14,16^. In perceptual terms, disruptive coloration interferes with feature binding^17^. In a similar way, iridescence creates changing colour and intensity boundaries, thereby disrupting the stable edge features normally used in object recognition: the brightness of iridescence may make (varying) parts of objects more conspicuous, but the changing colour patterns and boundaries could also deceive and confuse potential predators. This effect might be particularly acute in animals that lack the extensive upstream processing characteristic of the primate visual cortex.

Here, using artificial discs as prey and naïve *Bombus terrestris* as models for insect perception, we tested whether three forms of iridescence (multilayer and two types of diffraction grating: one cast from a flower, the other synthetic and perfectly regular) impair shape recognition. Bees are clearly not predators, but other hymenopterans are predators and parasitoids of many insects; furthermore, much is known about the bee visual system^18–20^, and they can easily be trained to perform discrimination tasks^21^.

## Results and Discussion

**Figure 1:**
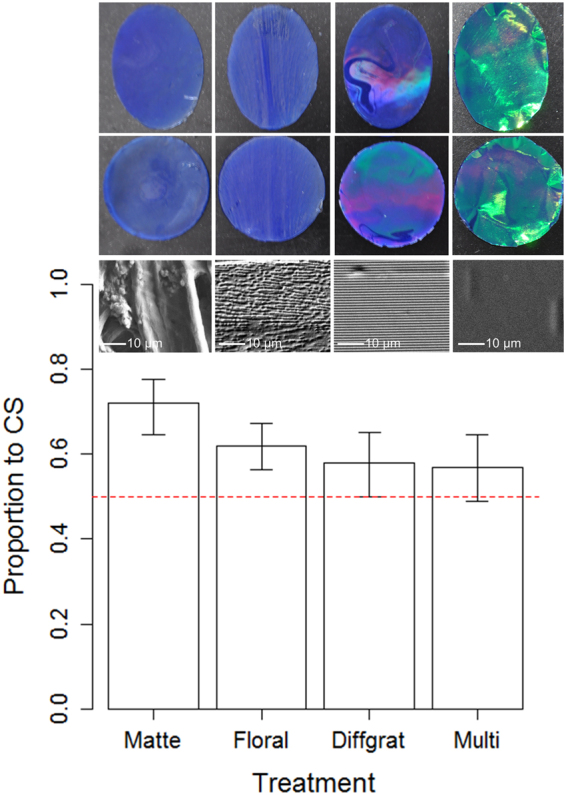
Experimental stimuli and results. Mean (±95% CI) proportion of visits to the conditioned stimuli for each treatment group, with corresponding photos (top two rows) and high magnification surface eSEM images (bottom row) of the four different treatment groups (from left to right; Matte, Floral diffraction grating, synthetic Diffraction grating and Multi-layer interference). The dashed line indicates the 50% expectation (i.e. the expected success rate for random foraging).

Using absolute conditioning^21^, we compared the recognition of oval and circular discs that displayed matte, floral diffraction grating iridescence, synthetic diffraction grating iridescence or multilayer iridescence on their surface (Fig. 1). All discs had equal surface areas and a blue ground colour (Fig. 1). For each treatment, 15 naïve bees were pre-trained to associate either an oval or a circular disc (randomly assigned for each individual and counter-balanced so half the test group were conditioned to one shape or the other) with a food reward. During this conditioning phase four discs of one shape and treatment (matte, floral diffraction grating, synthetic diffraction grating or multilayer iridescence that the bee would experience in the following experiment), each with a sucrose reward, were placed at random positions in the arena. Bees were individually released into the arena and a minimum of 10 visits (defined as drinking from the sucrose well in the disc) were recorded for each. The bees made on average 10.3 (range 10–13, *N* = 15/treatment group) visits before undergoing the experiment. After conditioning, the bees returned to the hive while the arena and discs were cleaned with ethanol to remove scent marks. For the experiment, which took place within minutes after the conditioning phase, a conditioned bee was released back into the arena, where eight unrewarded discs (four oval and four circular) had been randomly positioned. A total of 20 visits to the discs was observed for each bee. At this stage a visit was defined as either landing with all feet on the discs or drinking. The shape of the disc was recorded for each visit (see Material and Methods for details).

There was a significant effect of treatment on the bees’ ability to identify the previously rewarded stimulus (*χ*^2^ = 11.32, *d.f*. = 3, *P* = 0.0101). Tukey-type pair-wise comparisons showed that the bees had a stronger preference for this stimulus when targets were matte, compared to both the synthetic diffraction grating (*t* = 2.97, *P* = 0.0158) and multilayer iridescent targets (*t* = 3.11, *P* = 0.0098). However, there was no difference in discrimination when the matte and floral iridescent conditions were compared (*t* = 2.01, *P* = 0.1837), nor synthetic diffraction grating vs multilayer (*t* = 2.01, *P* = 0.1837), synthetic diffraction grating vs floral (*t* = 0.97, *P* = 0.7640) or multi-layer vs floral (*t* = 1.11, *P* = 0.6810). For matte (73% correct, *t* = 5.70, *P* = 0.0001), and floral iridescent targets (62% correct, *t* = 4.12, *P* = 0.0010) shape recognition was significantly higher than would be expected for random foraging, but this was not the case for targets with iridescence produced by synthetic diffraction gratings (58% correct, *t* = 1.93, *P* = 0.0748), or multilayer (57% correct, *t* = 1.70, *P* = 0.1117).

Our results demonstrate that iridescence impairs shape identification, in accordance with Thayer’s seemingly counter-intuitive proposal for a protective value of “*Brilliantly changeable or metallic colors”*. Both synthetic diffraction grating and multilayer iridescence impair shape recognition in bees, when compared to matte non-iridescent discs, suggesting that both strategies can be protective. Interestingly, the floral iridescence, where the iridescence is generated by disordered nanostructures that produces angle-dependent scattered light in a narrower range of wavelengths^4^ did not significantly impair the bees’ ability to discriminate shape. This difference in visual impact may explain why it is this subtle form of iridescence that has convergently evolved in several lineages of flowers^4^, rather than the more intense type of iridescence that leads to reduced discrimination. This suggests that functional aspects of iridescence, depending on the level of intensity, could be highly specific^22^.

We conclude that, just as it has deleterious effects on target tracking^23^ ‘strong’ iridescence, produced by multilayers or highly regular diffraction gratings, can act as camouflage by corrupting an otherwise recognisable shape. Future studies should investigate whether different types of iridescence have different functions and extend our results for insects to other species with different visual and cognitive systems.

## Material and Methods

### Target design

Following an established protocol^22,24^ the artificial targets were made of two components: (1) a colored disc made out of ‘2-Ton’ epoxy (Devcon, Danvers, MA, USA) and mixed with “ultramarine blue” color pigment (L. Cornelissen and Son, London, UK), and (2) a sucrose well made of an upturned lid of 1.5 ml Eppendorf containers and glued onto the disc.

We used Elite HD + Light Body silicone dental mould (Zhermack, Badia Polesine, Italy) to make the negative copies of the target discs, with equal amounts of base and catalyst. The matte copy was made by gluing a paper disc (“A4 Sketch Pad Paper”, W.H. Smith, London, UK) on top of a glass disc, and then gently push it down the dental mould with the paper-covered side of the glass disc faced downwards. The subtle “floral” diffraction-grating iridescent copy was made by gently pushing down a tulip (“Queen of the Night”) tepal in the dental mould. To make perfectly regular iridescence diffraction grating discs, the dental mould was instead cast from 1000 lines per mm unmounted holographic diffraction grating film (Edmund Optics, York, UK).

When the silicone dental mould mixture had hardened, the specimen was removed, leaving an inverted mould. The positive replicas of the floral iridescent, diffraction grating iridescent and matte discs were then made of the specimen by using the ‘2-Ton’ epoxy. Equal amounts of the resin and catalyst were combine with pigment (7 g resin + 7 g catalyst with 50 mg of “ultramarine blue”) and mixed. The inverted silicone mould was then filled with the pigmented epoxy. Once the cast was fully hardened (overnight at room temperature) it was removed from the mould. The multilayer iridescent targets were made by adding a sheet of multilayer thin film (Blue Iridescent Cellophane, Product code: AR01098, TTS, UK) on top of the epoxy resin immediately after pouring it into a dental mould cast by a glass disc (i.e. same as for the matte, but without the matte paper).

### Target measurements

**Figure 2:**
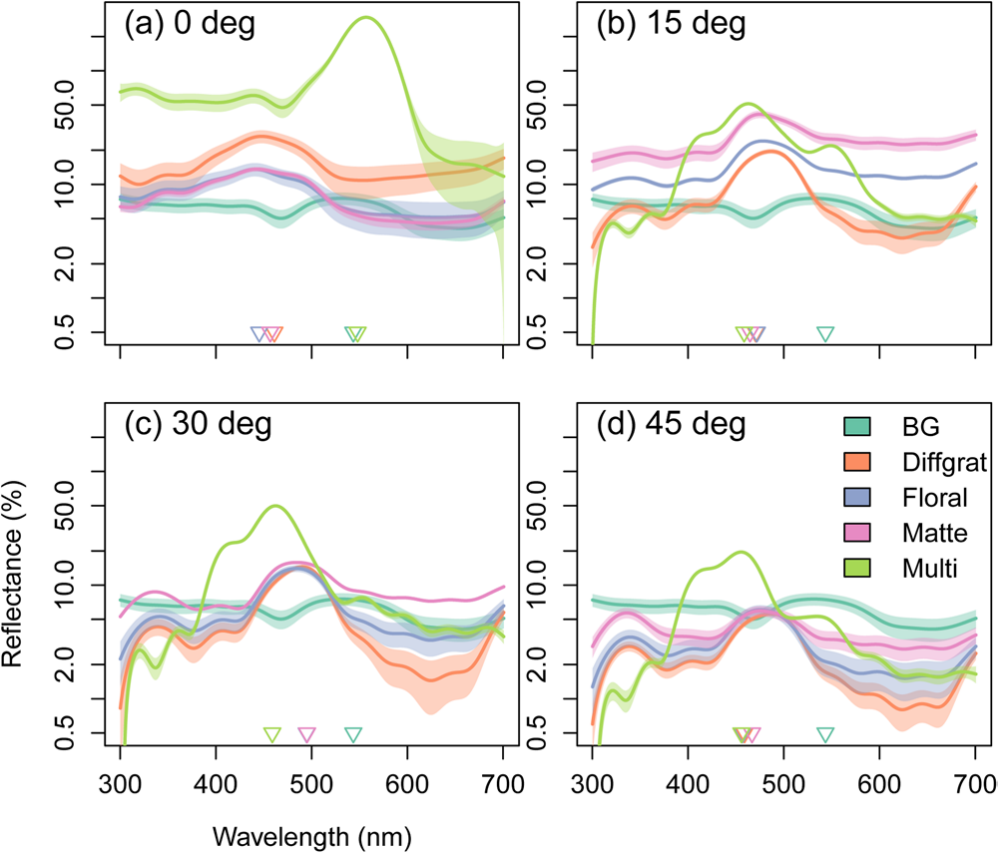
Reflectance spectra of the four treatment groups. Shown are estimated means and 95% CI from Generalized Additive Models with 20-degree-of-freedom spline smoothers. The triangles indicate the wavelength of maximum reflectance for each of the four treatment groups (each *N* = 9) at *θ* = 0°, 15°, 30° and 45°. The background (BG) is included in all graphs for comparison but was only measured at the relevant angle of *θ* = 0°.

Reflectance spectra of the four targets and of the bee arena background were measured using an Ocean Optics Flame-S- UV-VIS miniature spectrometer (Ocean Optics). A deuterium - halogen tungsten lamp (DH-2000-BAL UV-VIS-NIR, Ocean Optics) was used as a standardized light source, and measurements were taken using a premium-grade reflection probe (QR400-7-UV-VIS, Ocean Optics). We used a Stanley 1-77-153 FatMax CL2 Cross Line Laser to set the axis of the illuminating and reflection probe so that it was perpendicular (i.e. 90°) to the plane of the targets and background (BG) for targets at *θ* = 0°, and we also measured the targets, but not the background at *θ* = 15°, 30° and 45° (Fig. 2). A white spectralon standard (WS-1-SL, Ocean Optics) was used to calibrate the spectrometer. eSEM magnification images (Fig. 1) were taken of the surface structure of each of the four targets using a Zeiss EVO HD1 machine, at a working distance of 9 mm, EHT of 10 kV, and the I probe at 30 pA. Prior to the eSEM imaging, all samples were coated with 5 nm gold using a QUORUM Q150RES machine. For low magnification samples were at 800–808x mag, for high magnification samples were at 3.5 k–3.52 k x mag.

### Experimental design

Prior to the experiments, each bee (supplied by Syngenta Bioline, Little Clacton, Essex, UK) was individually marked using coloured Queen Marking Paints (EH Thorne, Beehives Ltd., Wragby, UK) for identification. Following the protocol of Whitney *et al*.^22,24^, each colony of bees were housed in plastic nesting boxes connected to a flight arena via a transparent plastic tube. The tube was regulated by a series of gates such that we could control the bees’ access to the flight arena. We used seven identical (l × w × h: 112 × 75 × 30 cm) wooden framed flight arenas topped with UV-transparent acrylic (Perspex^TM^) sheet for these experiments. The floor of each flight arena was covered in green AT 202 Advance Gaffa tape (Stage Electrics, Patchway, Bristol, UK). Illumination in the laboratory was provided by 46 Sylvania Activa 172 Professional 36 W fluorescent tubes (Osram Sylvania Inc., Wilmington, MA, USA) powered by Philips high frequency-ballasts (Philips NV, Amsterdam, Netherlands) with a flicker frequency higher than 20 KHz, on a 12:12 hour light/dark schedule. Temperature was automatically regulated and held between 21–23 °C. The bees were fed daily ad libitum with 30% sucrose solution and 3 times weekly with 15 g of pollen/colony. In order to motivate the bees to forage from the targets during the behavioural experiments, the sucrose solution was provided using wells (1.5 ml Eppendorf containers) fitted to epoxy targets of varying colours (albeit not the same as in the experiments). The flowers were randomly distributed inside the flight arena and redistributed each day so that the bees wouldn’t learn any specific positions of the flowers within the arena. For each behavioural experiment, a “rewarded” target means that it contained a 30% sucrose solution, whereas an unrewarded target means that it contained only water. Targets that were re-used in experiments were always cleaned with pure ethanol (Ethanol, absolute, >99.8% (GC), Sigma-Aldrich Ltd., St. Louis, Missouri, USA) to remove any possible scent marks from the bees^25^.

### Statistical analysis

All data were analyzed using R v. 2.13^26^. To test for the effect of target type (fixed effect with 4 levels: matte, floral iridescence, “perfect” iridescence produced by diffraction gratings and an iridescent multilayer) on the response variable “lands on conditioned and unconditioned stimuli” over the consecutive visits (20 in total), we used Generalized Linear Mixed Models (function glmer in the package lme4)^27^ to model the binomial error distribution. Individual bumblebee (*N* = 60) was treated as a random effect. We then carried out pair-wise comparisons between treatment groups with Tukey-type adjustment for multiple testing (function glht in the multcomp package)^28^. Finally, we tested whether the probability of landing on the pre-conditioned stimuli deviated from that expected by random choice (i.e. the expected value of 50%) for each treatment separately using Binomial GLMM’s.

### Data availability

The dataset supporting this article will be made available from the Dryad Digital Repository upon acceptance.
